# Disrupted cooperation between transcription factors across diverse cancer types

**DOI:** 10.1186/s12864-016-2842-8

**Published:** 2016-08-05

**Authors:** Jing Wang, Qi Liu, Jingchun Sun, Yu Shyr

**Affiliations:** 1 Center for Quantitative Sciences, Vanderbilt University School of Medicine, Nashville, TN USA; 2 Department of Biomedical Informatics, Vanderbilt University School of Medicine, Nashville, TN USA; 3 School of Biomedical Informatics, The University of Texas Health Science Center at Houston, Houston, TX USA; 4 Department of Cancer Biology, Vanderbilt University School of Medicine, Nashville, TN USA; 5 Department of Biostatistics, Vanderbilt University School of Medicine, Nashville, TN USA

**Keywords:** Transcription factors, TF cooperation, Cooperation disruption, Pan-cancer, Co-expression

## Abstract

**Background:**

Transcription Factors (TFs), essential for many cellular processes, generally work coordinately to induce transcriptional change in response to internal and external signals. Disrupted cooperation between TFs, leading to dysregulation of target genes, contributes to the pathogenesis of many diseases, including cancer. Although the aberrant activation of individual TFs and the functional effects have been widely studied, the perturbation of TF cooperativity in cancer has rarely been explored.

**Results:**

We used TF co-expression as proxy as cooperativity and performed a large-scale study on disrupted TF cooperation across seven cancer types. While the connectivity of downstream effectors, like metabolic genes and TF targets, were more or similarly disrupted than/with non-TFs, the cooperativity of TFs (upstream regulators) were consistently less disturbed in all studied cancer types. Highly coordinated TFs in normal, however, generally lost that cooperation in cancer. Although different types of cancer shared very few TF pairs with highly disrupted cooperation, the cooperativity of interferon regulatory factors (IRF) was highly disrupted in six cancer types. Specifically, the cooperativity of IRF8 was highly perturbed in lung cancer, which was further validated by two independent lung squamous cell carcinoma (LUSC) and lung adenocarcinoma (LUAD) datasets. More interestingly, the cooperativity of IRF8 was markedly associated with tumor progression and even contributed to the patient survival independent of tumor stage.

**Conclusions:**

Our findings underscore the far more important role of TF cooperativity in tumorigenesis than previously appreciated. Disrupted cooperation of TFs provides potential clinical utility as prognostic markers for predicting the patient survival.

**Electronic supplementary material:**

The online version of this article (doi:10.1186/s12864-016-2842-8) contains supplementary material, which is available to authorized users.

## Background

Transcription factors (TFs) are proteins that bind to either promoter or enhancer regions of a gene, thereby regulating the transcription activity of the gene [1, 2]. About 10 % of genes in the human genome encode TFs and they are essential for many cellular processes [3]. A high proportion of TFs either acts as oncogenes or tumor suppressors or regulates the activity of pathways related to tumor progression. The deregulation of these TFs, reshaping the expression of their target genes, leads to tumor formation, progression, and metastasis [4]. Numerous studies have revealed the functional impact of aberrant activation of individual TFs on tumor progression. For example, mutations of *BRCA1* and *BRCA2* cause the genetic instability of the cell and thus confer a substantial risk of breast and ovarian cancer [5].

Rather than function alone, transcription factors generally cooperate to control gene expression. Like aberrant activation of individual TFs, disruption of TF cooperativity also alters the expression of downstream genes and contributes to disease pathogenesis. Several studies have reported the alteration of TF cooperativity in cancer [6–15]. For example, the association between SNAIL and ZEB1 is lost in human colon carcinomas [16]. SNAIL, ZEB, and basic helix-loop-helix (bHLH) factors ally to mediate the dynamic silencing of CDH1, and loss of CDH1 function is a hallmark of carcinoma cell invasiveness [17, 18]. Three transcription factors TTF1/NKX2-1, NKX2-8, and PAX9 show pronounced synergy in promoting the proliferation of immortalized human lung epithelial cells, and the alteration of their cooperativity contributes to lung cancer development [19]. Compared with the rapid accumulation of knowledge on individual TFs, however, there is only limited research on TF cooperativity and our understanding of TF cooperativity perturbation in cancer is lagging far behind.

Large-scale genomics projects, such as The Cancer Genome Atlas (TCGA), generating various omics data for thousands of tumors with matched normal samples [20], have provided a great opportunity to explore the common and specific disruption of gene co-expression across multiple cancer types. For example, West et al. has demonstrated that cancer is characterized by an increase in network entropy, i.e., reduced absolute gene correlations, while cell cycle/proliferation genes are preferentially associated with significant reductions in network entropy [21]. Here we focused on transcription factors, which are upstream regulators of transcriptome change that results in phenotypic change. We analyzed the dysregulated cooperation between TFs across seven cancer types and compared that with the dysregulation between non-TFs and other downstream effectors. We not only identified specific TF pairs highly disrupted in each individual cancer type but also detected common TFs whose cooperativity were significantly disturbed across diverse cancer types. Finally, the disrupted cooperation of IRF8 in lung cancer was validated by two independent lung squamous cell carcinoma (LUSC) and lung adenocarcinoma (LUAD) datasets, and its contribution to tumor progression and patient survival was further investigated.

## Results and discussion

### TF cooperativity is less disturbed than non-TF in cancer

We obtained 1991 human TFs in total by combining three databases, AnimalTFDB [22], TRANSFAC [23], and TFCat [24] (Methods). We studied seven cancer types and each cancer type had mRNA expression profiles measured for both tumor and matched normal samples (Table 1).

**Table 1:** Sample size for each cancer type

	CancerType	Normal-tumor pair	Source
Discovery	NSCLC	85	GSE32665
	PRAD	58	GSE6919
	COADREAD	32	GSE8671
	BRCA	61	GSE14999
	HNSC	41	TCGA
	KIRC	72	TCGA
	LIHC	50	TCGA
Validation	LUAD	57	TCGA
	LUSC	50	TCGA

TF co-expression has been commonly used to predict TF cooperativity [25–27]. For instance, Hammonds et al. used TFs co-expression to suggest their co-association [25]. Zhou et al. developed a second-order expression similarity to infer TFs’ cooperativity [27]. Here we used two lists of known cooperative TFs to study the relationship between co-expression and cooperativity. One included interacting TFs with high confidence from HitPredict (http://hintdb.hgc.jp/htp/) [28] , and the other contained TF co-associations based on the non-randomly distributed TF binding regions from ENCODE [29]. We used Spearman correlation coefficient to measure the co-expression, which is more robust to outliers than Pearson correlation. Compared to random TF pairs, cooperative TFs were more tightly co-expressed in all the studied cancer types (Wilcoxon Rank Sum test, *p* < 1e-4) (Additional file 1: Figure S1). Among 712 co-associated TFs in the HepG2 cell line, even higher correlations were observed in the corresponding liver cancer (LIHC) than general TF associations and random TF pairs (Additional file 1: Figure S1). These findings demonstrated that TF co-expression can act as an appropriate proxy for cooperativity.

There was no general bias for TFs on the measure of cooperativity compared to other genes. The scatterplot containing the pair-wise correlation coefficient on the y-axis and the average expression of genes (log_2_ scale) on the x-axis generally followed a horizontal line around zero in all tumor and normal samples (Additional file 2: Figure S2), suggesting no intensity-dependent bias on the measure of cooperativity. TFs had comparable concentrations with non-TFs in each individual cancer type and matched normal samples as well (Additional file 3: Figure S3). In addition, TFs, non-TFs and TF targets showed similar cooperativity level in all types of matched normal samples (Additional file 4: Figure S4).

To measure the alteration of cooperation, we used the absolute correlation change in tumor versus normal (Methods). Analysis of gene correlation change, i.e., differential co-expression analysis, is a more comprehensive technique to the differential expression analysis [30, 31]. Correlation change between TFs is not only caused by expression alteration of individual TFs, but also driven by subtle perturbation in TFs expression coordination, which both potentially leads to the dynamic switch of TF partners [32, 33].

**Fig. 1:**
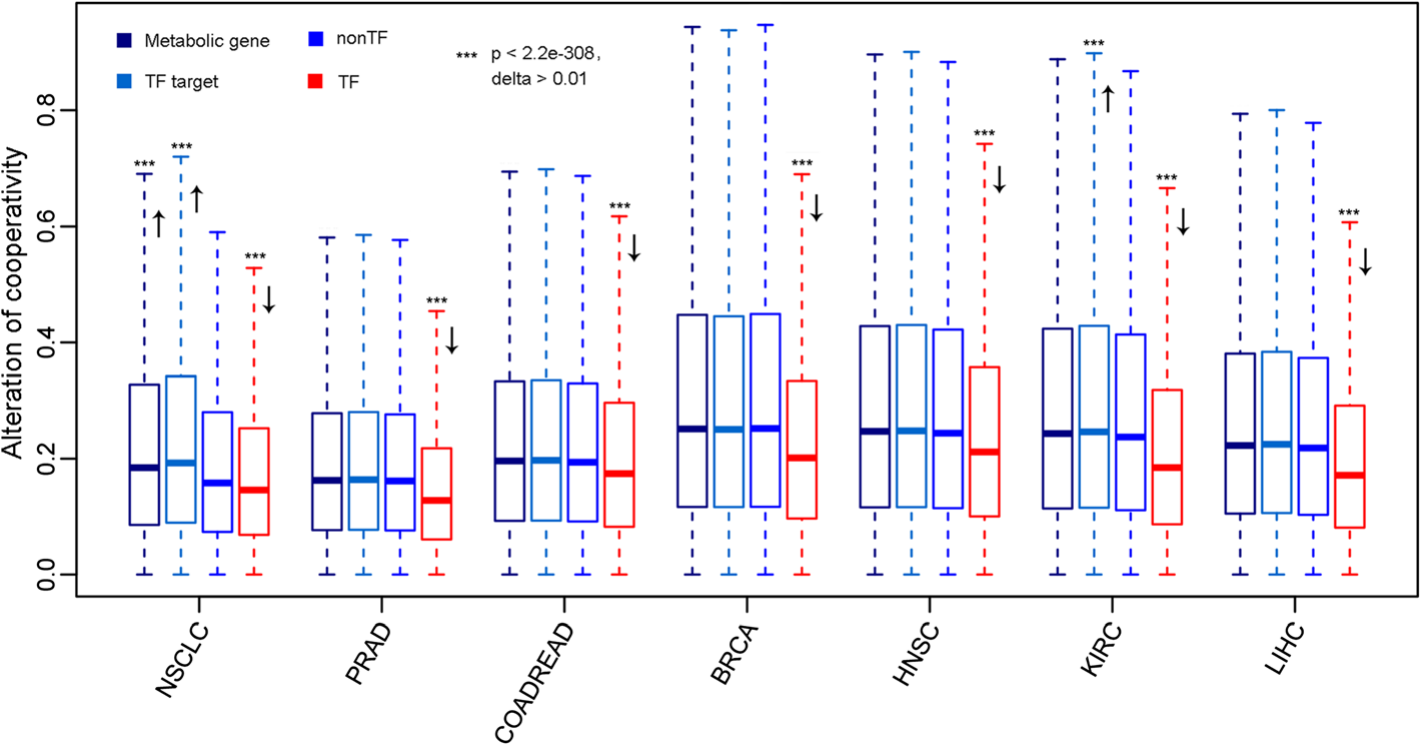
Cooperativity alterations in tumor versus normal. The box plots illustrate the absolute correlation change in tumor relative to normal between TF pairs (*red*), non-TF pairs (*blue*), metabolic genes (*steel blue*) and TF target genes (*light blue*) in NSCLC, PRAD, COADREAD, BRCA, HNSC, KIRC, and LIHC

Although TF and non-TFs showed comparable cooperative level, the absolute correlation change between TFs was significantly smaller than that between non-TFs across all studied cancer types (Wilcoxon Rank Sum test, *p* < 2.2e-308; difference between the medians (delta) >0.01) (Fig. 1). Specifically, breast invasive carcinoma (BRCA) exhibited the largest difference of correlation change in TFs compared to non-TFs, followed by kidney renal clear cell carcinoma (KIRC), liver hepatocellular carcinoma (LIHC), and non-small-cell lung carcinoma (NSCLC) (Fig. 1). In contrast, two other groups of genes, metabolic genes and TF target genes, did not show the consistent smaller correlation changes like what TFs presented across different cancer types. Instead, these two groups of genes either showed larger or same correlation change compared to those of non-TFs (Fig. 1). The fact that only the cooperation between TFs is significantly less disturbed than non-TFs in all types of tumor relative to normal tissues could be explained by the functional roles of transcription factors rather than statistical artifacts or other confounding factors. As upstream regulators for determining gene expression, the cooperativity between TFs is considerably constrained and more robust to disease than that of downstream effectors, like TF targets or genes in metabolic pathway [34]. Restricting our analysis on two lists of known cooperative TFs, however, we didn’t observe the consistent constrained TF cooperativity across diverse cancer types, which was possibly due to the low coverage and bias of existing knowledge [35, 36]. There are only 114 TFs with predicted associations from ENCODE, which covers only 5.7 % of known TFs. Although there are much more TFs from HitPredict compared to ENCODE, they have similar number of TF interactions, which suggests the number of TF interactions of HitPredict is far less than expected. Moreover, protein-protein interaction networks are generally associated with multiple types of biases, e.g., selection preference (disease proteins) and technical limitations [35, 36].

### Tightly coordinated TFs lost cooperation in cancer

**Fig. 2:**
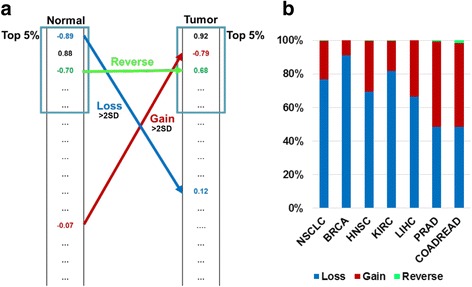
The pair-wise TF cooperativity is mainly lost in tumor relative in normal. **a** The schema of TF cooperation change. TF pairs with significant coordination changes (>2SD) that only appeared in the highly coordinated list in tumor but not in normal were defined as “Gain”. TF pairs with significant coordination changes (>2SD) that only presented in the highly coordinated normal list were defined as “Loss”. TF pairs showing both in the tumor and normal list but opposite direction (e.g., positively correlated in tumor but inversely correlated in normal or vice versa) were classified as “Reverse”. **b** Bar plot depicts percentage of Loss, Gain and Reverse in TF cooperativity alteration

Although the cooperativity between TFs is considerably constrained during tumorigenesis, some TF pairs significantly changed their coordination in cancer. We focused on TF pairs tightly coordinated in either normal or tumor samples (top 5 % here). We tracked the cooperation changes of these TF pairs and classified them into three categories, “Gain”, “Loss” and “Reverse” (Fig. 2a). TF pairs with significant coordination changes (>2SD) that only appeared in the highly coordinated list in tumor but not in normal were defined as “Gain”. Accordingly, TF pairs with significant coordination changes (> 2SD) that only presented in the highly coordinated normal list were defined as “Loss”. TF pairs showing both in the tumor and normal list but opposite direction (e.g., positively correlated in tumor but inversely correlated in normal or vice versa) were classified as “Reverse”. As expected, there were very few TF pairs belonging to the “Reverse” category across all studied cancer types. In most types of cancer, “Loss” is dominant over “Gain”, especially in BRCA, NSCLC and KIRC (Fig. 2b). Due to the dominant effect of losing tightly coordinated TF pairs rather than gaining during tumorigenesis, highly coordinated TF pairs in tumor were less disturbed than those in normal. In BRCA, the highly cooperative TFs in tumor were even less altered than other TFs (Additional file 5: Figure S5). Consistent with previous studies revealing cancer is globally characterized by reduced absolute gene correlations [21], our results indicate that tightly coordinated TFs losing their normal cooperation play a major role in tumorigenesis. That is, disrupting the normally coordinated TFs affect gene expression of their target genes, leading to tumor initiation and progression.

### Highly dyscooperated TFs across diverse cancer types

**Fig. 3:**
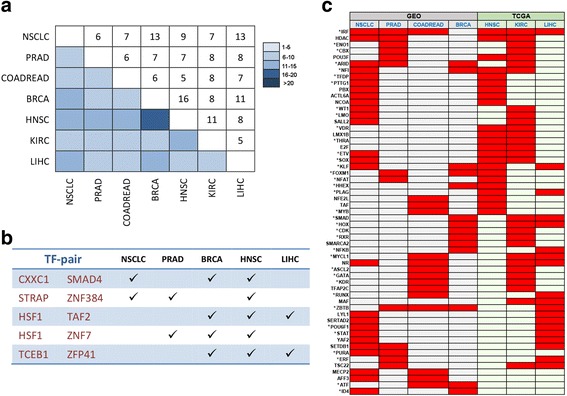
Highly disrupted TFs across diverse cancer types. **a** Heat map of highly disrupted TF pairs shared across different cancer types. Upper triangle shows the number of common pairs. **b** A list of highly disrupted TF pairs shared by more than two cancer types. **a** and **b** are based on the top 3000 TF pairs with the highest disruption in cooperativity. **c** Heat map of common highly disrupted TFs across different cancer types. Red bars designate that the TF labeled on the left is identified in the cancer type labeled on the top with greatly disturbed cooperativity with other TFs. Datasets from GSE and TCGA are shaded in different colors. TFs marked with “*” function association with tumor progression

We ranked the disrupted TF pairs in each type of cancer, trying to identify the TF pairs whose cooperativity is commonly disturbed across diverse cancer types. We found different cancer types shared very few highly disrupted TF pairs. Among the top 3000 TF pairs with the highest disruption in cooperativity, there were only 5 ~ 16 TF pairs shared between two cancer types (Fig. 3a). Moreover, there were only 5 TF pairs shared by three or more cancer types (Fig. 3b). The correlation plots of these 5 TF pairs and correlation measures in normal and tumor were given in Additional file 6: Figure S6. Consistent with our previous finding, most of TF pairs lose cooperation during tumorigenesis, such as CXXC1-SMAD4 in NSCLC and BRCA. We also observed that few uncorrelated TFs in normal became co-expressed in tumor, e.g., HSF1-ZNF7 in BRCA and HNSC (Additional file 6: Figure S6), indicating abnormal co-expression potentially drive tumorigenesis as well. *TAF2* and *HSF1* were identified as driver genes of liver carcinoma and closely linked on chromosome 8q24 [37, 38]. This finding reveals that tumor is quite different from each other on disrupted TF pairs. Among the top 3000 TF pairs with the highest disruption in cooperativity in each cancer type, less than 50 % of TF pairs were due to the differential expression of TF itself except KIRC (62.3 %). Among the 5 common TF pairs, only the disruption of TCEB1-ZFP41 in LIHC was caused by the differential expression of ZFP41 (|log_2_FC| > 1 & FDR < 0.05).

We aggregated the disrupted cooperation of TF pairs to each individual TF by calculating the number of significantly changed cooperation for this TF (Methods). The TFs with significant number of broken cooperative relationship are called “highly dyscooperated” TFs. As a result, more than 20 highly dyscooperated TFs were identified for each cancer type. KIRC identified 87 highly dyscooperated TFs, and NSCLC discovered 61, and 44, 42, 34, 28 and 24 were recognized for LIHC, HNSC, COADREAD, PRAD, and BRCA respectively (Additional file 7: Table S1). Less than 50 % of these dyscooperated TFs were differentially expressed in the corresponding cancer type (|log_2_FC| > 1 & FDR < 0.05). Compared with the low overlap of disrupted TF pairs, the dyscooperated TFs were much more common between different types of cancer, which either share the same TFs or TFs belonging to the same family. For example, NSCLC shared 47.5 % of its highly dyscooperated TFs/TF families with other cancer types (Fig. 3c). Notably, there were 57 TFs/TF families common in two or more cancer types (Fig. 3c). These highly disrupted TF families included well-known genes related to tumor progression, such as the *HOX* family, the *ZBTB* family, *WT1*, and so on [39–41]. Most interestingly, the interferon regulatory factors (IRF) family was detected in six out of the seven studied cancers. Specifically, IRF8 was identified as one of the most dyscooperated TFs in NSCLC and COADREAD, IRF6 was detected in PRAD, IRF5 was found in HNSC and KIRC, and IRF2 was discovered in LIHC (Fig. 3c). IRF family members share a DNA-binding domain (DBD) and recognize a consensus motif, 5′-AANNGAAA-3′ [42]. Previous studies have revealed the versatile and critical functions performed by the IRF family transcription factors, including immune response, cell growth regulation, cell apoptosis, and hematopoietic development [43–45]. Moreover, IRF family members contribute to tumorigenesis and tumor progression. For example, *IRF6* interacts with maspin (mammary scrine protease inhibitor), which is characterized as a tumor suppressor [46]. The targets of IRF8 are involved in anti-apoptotic function [47]. Reported as a direct target of *p53* [48], *IRF5* can also regulate the cell cycle and apoptosis in a *p53*-independent way [49, 50]. *IRF2* is an oncogenic gene which plays a positive role in the cell cycle regulation of the human histone H4 gene *FO108* [51]. Taken together, it can be inferred that the dyscooperated IRF family might be a common mechanism in tumor initiation or progression.

### Functional effect of dyscooperated IRF in tumorigenesis

GO enrichment analysis was performed for the target genes of IRF transcription factors, which were identified by motif searching and further filtered by chromatin accessibility from DNase-seq data in the corresponding cell lines, to investigate their potential functional roles. The target genes are enriched in many cancer related functions such as cell death, response to stress, cell proliferation, and regulation of apoptotic processes. Furthermore, 132 (accounting for 25.3 %) genes are annotated as cancer census genes in the Cancer Gene Census of COSMIC [52]. Among them, *BRAF* is an important gene associated with various cancers, including colorectal cancer, malignant melanoma, thyroid carcinoma, non-small cell lung carcinoma, and adenocarcinoma of lung [53, 54]; *NDRG1* acts as a tumor suppressor gene involved in stress responses, cell growth, and differentiation, and its expression may be a prognostic indicator for several types of cancer [55–57].

**Fig. 4:**
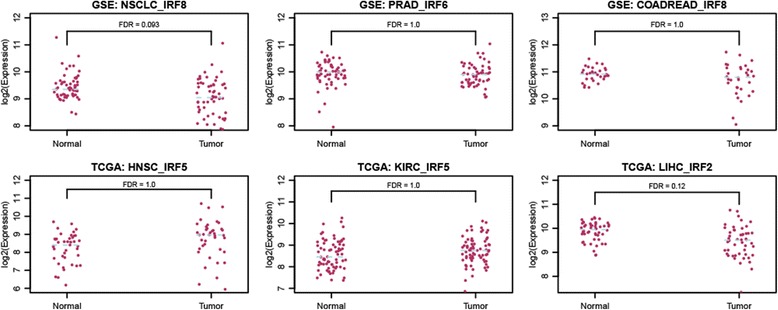
Expression abundance of IRF in tumor versus normal. Each point in the plot represents a sample. FDR is given on the top, and the median concentration in tumor/normal is shown by a dashed line

**Fig. 5:**
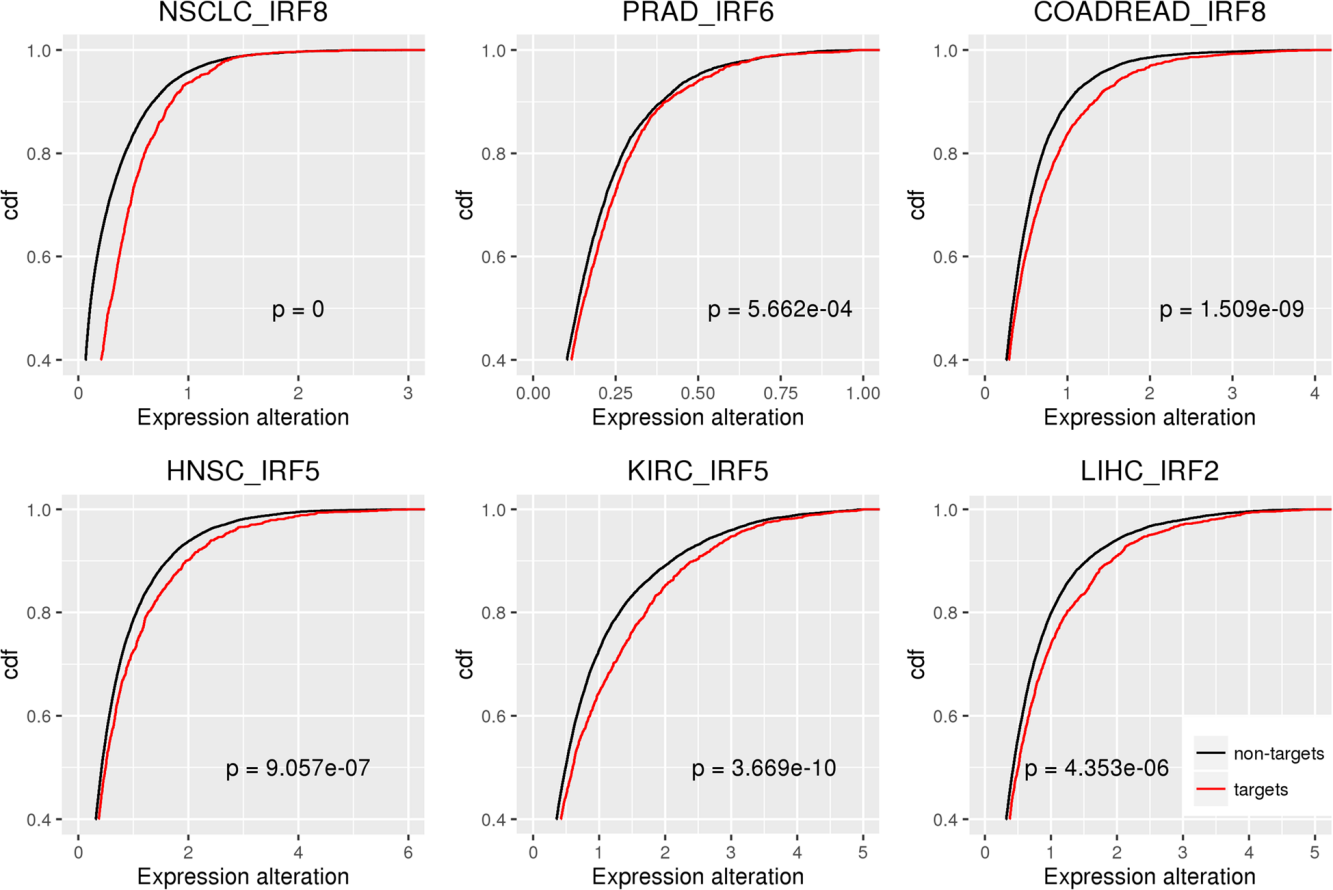
Comparison of expression alterations between IRF’s targets and other genes. The plots illustrate the cumulative distribution function (cdf) of expression alteration in tumor versus normal for IRF’s targets and non-targets across six cancer types. The x-axis is the absolute value of log_2_ fold change of expression. The significance of difference between target and non-target genes is also shown in the plots

We explored the potential cause and functional effect of dyscooperated IRF family transcription factors in the six cancers. We first compared the expression abundance of IRF transcription factors in tumor versus normal to see whether the dyscooperation is caused by differential expression of the TF itself. We found that the IRF transcription factor was not significantly differentially expressed in each tumor versus the matched normal (Fig. 4), which means the dyscooperation of IRF members was caused by perturbation in expression coordination rather than expression alteration of the IRF itself. Moreover, the expression abundances of IRF target genes were more significantly disturbed than non-target genes (Kolmogorov–Smirnov test, the *p*-value in NSCLC, PRAD, COADREAD, HNSC, KIRC and LIHC is 0, 5.66e-4, 1.51e-9, 9.06e-7, 3.67e-10 and 4.35e-6 respectively) (Fig. 5). To reduce the potential bias introduced by the global effect of tumorigenesis, we further compared the expression alteration of IRF’s targets and non-targets involved in the stress response. As a result, the targets involved in stress response were more likely to be dysregulated than non-targets with the same function across all six cancer types (Additional file 8: Figure S7). These results demonstrated that disturbance of the IRF cooperativity affects the transcription of downstream target genes. The observation that the dysregulation of the target genes is mainly caused by the disrupted TF cooperation rather than differentially expressed TF itself suggests that the analysis of TF cooperativity in tumor is more important than previously recognized. It holds great potential for identifying novel drivers that could never be discovered by regular differential expression analysis.

### Validation of dyscooperated IRF8 using LUAD and LUSC datasets

**Fig. 6:**
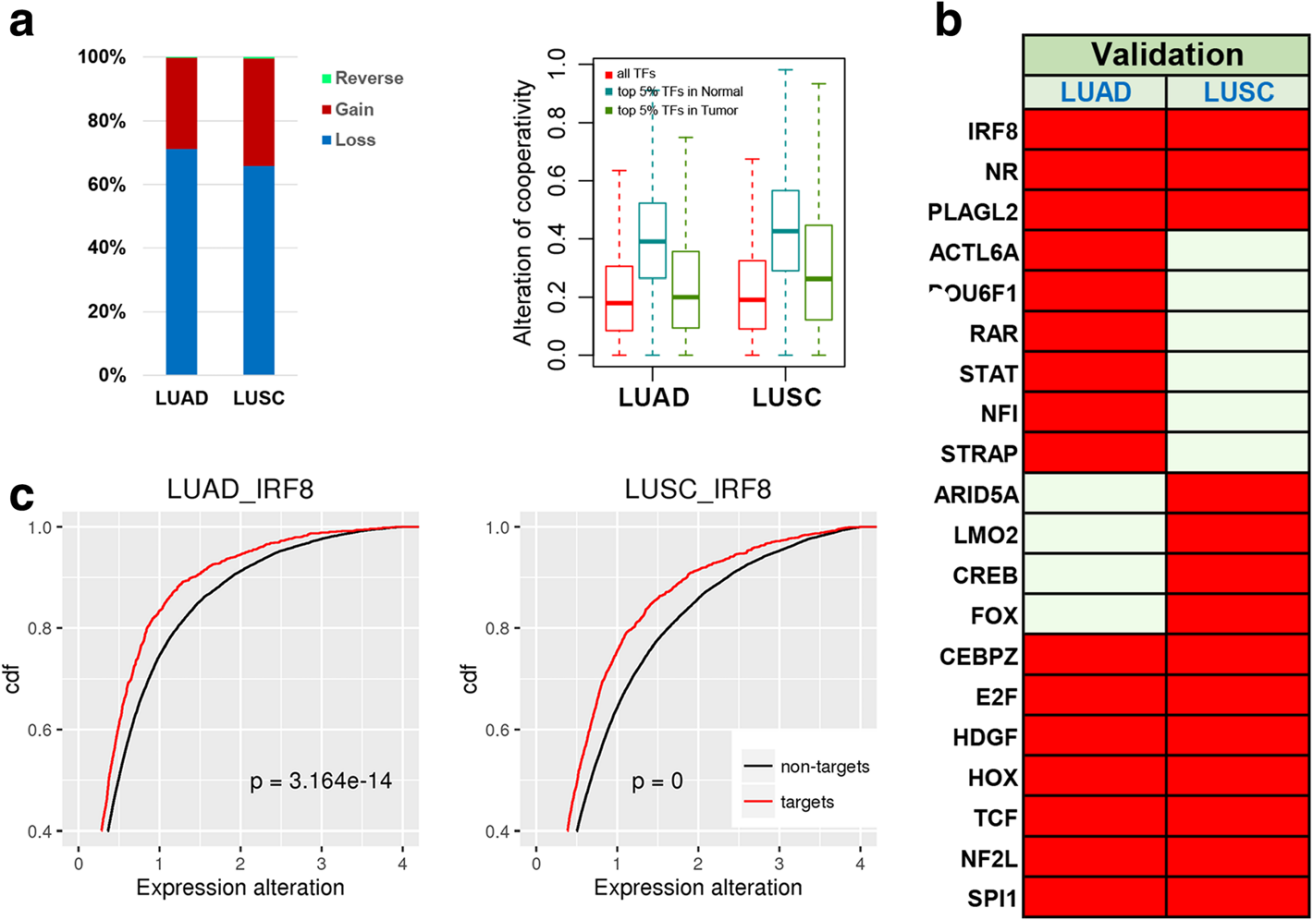
Validation of dyscooperated IRF8 in LUAD and LUSC. **a** Cooperativity alterations between TF pairs that highly coordinated in normal/tumor. **b** Highly dyscooperated TFs also identified in the previous NSCLC dataset. **c** Comparison of expression alteration between IRF8’s target and non-target genes

We downloaded two public RNA-seq datasets from TCGA and performed the same analysis on the disrupted TF cooperation. One includes 57 LUAD patient tumor and adjacent normal samples, and the other consists of 50 LUSC tumor and matched normal samples (Table 1). Consistent with our observation in the discovery cohort, we found TFs had comparable expression abundances with non-TFs in both LUAD and LUSC datasets. Highly coordinated TF pairs dominantly lost cooperativity during tumorigenesis, and those highly coordinated TF pairs in tumor were less disturbed than those in normal (Fig. 6a). We further identified dyscooperated TFs in these two datasets. The results showed that 13 out of the 61 identified highly dyscooperated TFs in the discovery NSCLC cohort were confirmed in the LUSC and/or LUAD datasets (Fig. 6b). The IRF family member, IRF8, was confirmed in both LUAD and LUSC datasets. Moreover, seven additional highly dyscooperated TFs were detected in both LUAD and LUSC (Fig. 6b). Additionally, the expression of IRF8’s targets was more significantly altered than non-targets in tumor versus normal (Kolmogorov–Smirnov test, p-value < 3.16e-14, Fig. 6c). Repeated studies on the two independent datasets reproduced the findings from the discovery cohort, which demonstrate that the disruption of TF cooperativity is highly reproducible and biological meaningful in cancer.

**Fig. 7:**
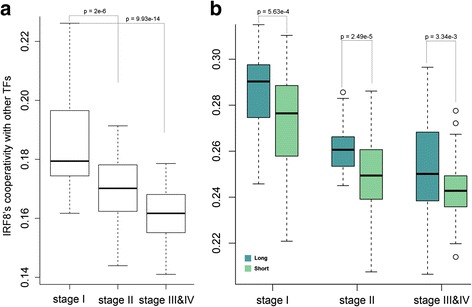
The IRF8 cooperativity associated with tumor progression and patient survival. **a** IRF8 loosens its cooperation with other TFs along with cancer progression in LUSC. **b** IRF8 is more loosely coordinated with other TFs in patients with poor survival within each LUSC stage

Tumors progress through a series of stages, which is the most important factor in prognosis. We divided the LUSC patients into different groups based on tumor stages and aggregated the cooperativity of IRF8 in each individual group. We combined stage III and IV together since there are only few samples in stage IV. As a result, we have 211 stage I, 123 stage II, and 82 stage III/IV samples, respectively. Notably, we found that the cooperativity of IRF8 was remarkably inversely correlated with tumor stages. As the tumor stage increasing, IRF8 cooperation with other TFs became looser and looser (Fig. 7a), which indicates that IRF8 cooperativity might play an important role in cancer development. More importantly, we investigated the survival contributions of IRF8 cooperativity within each LUSC stage. We divided patients into two groups according to survival time, long-survival and short-survival. We found that IRF8 was more tightly cooperated with other TFs in patients with long survival than those with short survival within the same tumor stage (Fig. 7b, Wilcoxon Rank Sum test, the p-value is 5.63e-4, 2.49e-5, and 3.34e-3 for stage I, II, and III&IV patients respectively). That is, the cooperativity of IRF8 contributes to the survival of patients independent of tumor stage. These results demonstrated that the disruption of TF cooperation provides clinical utility for predicting patient survival.

## Conclusions

We characterized the disrupted cooperation of transcription factors in cancer and identified common TFs whose cooperativity was highly disrupted across diverse cancer types. Our findings underscore the far more important role of TF cooperativity in tumorigenesis than previously recognized. Disrupted cooperation of TFs provides potential clinical utility as prognostic markers for predicting the patient survival.

## Methods

### Datasets

The gene expression profile analysed in this study included both next-generation sequencing (NGS) and microarray data. For the seven cancer types for discovery analysis, the mRNA-seq data of HNSC, KIRC, and LIHC were downloaded from Firehose developed by the Broad GDAC (https://confluence.broadinstitute.org/display/GDAC/Dashboard-Stddata). The microarray data for NSCLC (GSE19804), PRAD (GSE6919), COADREAD (GSE8671), and BRCA (GSE14999) were downloaded from the Gene Expression Omnibus (GEO, http://www.ncbi.nlm.nih.gov/geo/) [58]. The two independent datasets for validation, LUAD and LUSC, and the clinical data were also obtained from Firehose. The detailed information about the sample size and source for each dataset is shown in Table 1. We excluded genes if they were not expressed in more than 50 % of samples for TCGA RNA-seq data. For affymetrix microarray data, we applied data-dependent cutoffs to remove lowly expressed genes based on their average signal (log_2_ signal intensity <7 for GSE32665, log_2_ signal intensity <2 for GSE6919 and GSE8671).

After combining 1469 TFs in AnimalTFDB (v2.0) [22], 1837 in TRANSFAC (v2014.1) and 405 in TFCat [23, 24] and removing redundant TFs, we obtained 1991 human TFs in total (Additional file 9: Figure S8). The metabolic genes were downloaded from UniProt-GOA (release 2014_11, GO: 0008152) [59], and the TF target genes were downloaded from MSigDB (v5.0, C3: motif gene sets, TFT: transcription factor targets) [60].

### Disruption of cooperativity between genes

We used co-expression as proxy for cooperativity. Spearman correlation coefficients were used to measure the correlation between any pair of genes, which is more robust to outliers than Pearson correlation. The absolute correlation change of any pair of genes in tumor relative to normal was used to measure the alteration of cooperativity. The cooperativity alteration between gene *i* and *j* was defined as:

1 $$ \left|{C}_{ij}^{T-N}\right| = \left|{C}_{ij}^T - {C}_{ij}^N\right| $$

where *C*_*ij*_^*T*^ is the correlation between gene *i* and *j* in tumor, and *C*_*ij*_^*N*^ denotes the correlation in matched normal tissue. We compared the cooperativity alteration between TF pairs (*i* ∈ *TF & j* ∈ *TF*, j ≠ *i* ), gene pairs in metabolic pathway, and TF target pairs with non-TF pairs (*i* ∉ *TF & j* ∉ *TF*) using the Wilcoxon Rank Sum test [61]. We used the criteria that the median difference is greater than 0.01 and the p-value is less than 1e-20.

To assess the disruption of the cooperation of individual TF *i* in cancer *C*_*i*_^*T* − *N*^, we aggregated the cooperativity alterations of this TF with other TFs. The cooperativity alteration between TF *i* and other TFs *j* (j ≠ *i* , *j* ∈ *TF*) was z-transformed and the number of significantly changed cooperation, positive change or negative changes, was counted separately,

2 $$ {C}_i^{T-N}\to \Big\{\begin{array}{c}{\displaystyle \sum_{j=1,j\ne i}^n}{P}_{ij}\kern0.1em ,\kern1.85em  if\kern0.2em {Z}_{C_{ij}^{T-N}}>3{P}_{ij}=1; otherwise\ {P}_{ij}=0\kern1em \\ {}\kern1em {\displaystyle \sum_{j=1,j\ne i}^n}{N}_{ij}\kern0.1em ,\kern1.20em  if\kern0.2em {Z}_{C_{ij}^{T-N}}<-3\kern0.5em {N}_{ij}=1; otherwise\ {N}_{ij}=0\end{array}\kern1em \operatorname{} $$

where *n* is the number of TFs, and ${Z}_{C_{ij}^{T-N}}$ is the z-score of the cooperation change between TF *i* and *j* in tumor versus normal. The change is considered significant if the z-score is greater than 3 or less than -3. We identified highly disrupted TFs by setting the cutoffs. The cutoffs were chosen where the decreasing number of highly disrupted TFs was less than or equal to 10 as the increasing requirement of the number of significantly changed cooperation (Additional file 10: Figure S9).

### Statistical analysis

The gene expression abundances were log_2_ transformed and the paired t-test was used to identify differentially expressed genes between tumor and matched normal tissues. The Kolmogorov–Smirnov test was used to compare the expression changes between IRF’s targets and non-targets, and between IRF’s targets and non-targets involved in stress response. The Wilcoxon Rank Sum test was applied to compare the cooperativity alteration of TF pairs, metabolic gene pairs, and TF target pairs with that of non-TF pairs. The BH method was used to adjust p-values for multiple testing. All statistical tests in this study were implemented by R (version 3.0.3).

### IRF targets identification and GO enrichment analysis

Match program (version 1.0) [62], provided by TRANSFAC database, was used to identify putative IRF binding sites in the promoter regions, which were defined as 500bp upstream and 100bp downstream of the transcription start site. The characteristic motifs for binding sites searching were obtained from TRANSFAC and listed in Table 2. Motif hits were further filtered by only considering those that fall in chromatin accessible regions for each type of cancer, which were obtained from ENCODE DNase-seq data (Additional file 11: Table S2). For example, we identified 1546 IRF8’s targets in NSCLC and 431 of them are involved in the function of stress response (Additional file 12: Table S3). The GO enrichment analysis for IRFs targets was performed via WebGestalt [63]. Functional categories with FDR <0.0001 were reported.

**Table 2:** Motifs for binding sites searching

Motif	Matrix_acc^a^	IRF2	IRF5	IRF6	IRF8
5′-RAAANTGAAAN-3′	M00972	✔	✔		✔
5′-BNCRSTTTCANTTYY-3′	M00772	✔	✔	✔	✔
5′-GAAAAGYGAAASY-3′	M00063	✔			
5′-RAARTGAAACTG-3′	M00699				✔

^a^Matrix_acc means the accession number of matrix in TRANSFAC

### The cooperation of IRF8 associated with clinical outcome

We studied the relationship between the IRF8 cooperativity and clinical outcome on LUSC dataset, which provided relatively more complete clinical information than the LUAD dataset. In total, 416 LUSC patients were included, among which, 211 were classified as stage I, 123 as stage II, and 82 as stage III/IV. We quantified the cooperativity of IRF8 by summarizing its cooperation score with other TFs,

3 $$ {C}_{IRF8}={\displaystyle \sum_{j=1,T{F}_j\ne IRF8}^n}{C}_{IRF8,j} $$

where *C*_*IRF*8,*j*_ denotes the cooperation of IRF8 with TF *j*, and n is the number of TFs. To make the cooperativity of IRF8 comparable between stages, we applied the subsampling technique. We selected 80 % of 82 patients (the smallest sample size of LUSC stages) and calculated the cooperativity of IRF8 in each resampling dataset, and we ran 50 resamplings. We compared the distribution of cooperativity of IRF8 between different stages and found that IRF8 has the highest cooperativity in stage I but the lowest cooperativity in stage III/IV.

To further investigate the contribution of IRF8 cooperativity to patient survival independent of tumor stage, we classified patients into two groups by their survival time: long-survival (top 25 %) and short-survival (bottom 25 %) within each stage. We compared the IRF8’s cooperativity between these two groups.

## Abbreviations

bHLH, basic helix-loop-helix; BRCA, breast invasive carcinoma; COADREAD, colorectal adenocarcinoma; GEO, Gene Expression Omnibus; HNSC, head and neck squamous cell carcinoma; IRF, interferon regulatory factor; KIRC, kidney renal clear cell carcinoma; LIHC, liver hepatocellular carcinoma; LUAD, lung adenocarcinoma; LUSC, lung squamous cell carcinoma; NGS, next-generation sequencing; NSCLC, non-small-cell lung carcinoma; PRAD, prostate adenocarcinoma; RS, response to stress; TCGA, The Cancer Genome Atlas; TF, transcription factor.

## Additional files

Additional file 1: Figure S1. The correlation between TF pairs from different datasets. The box plots illustrate the correlation (absolute value of Spearman correlation coefficient) between interacted TFs from HitPredict (HitPredict-TF), associated TFs and HepG2-specific associated TFs from ENCODE (ENCODE associated-TF), and the correlation of random TF pairs (random TF) in NSCLC, PRAD, COADREAD, BRCA, HNSC, KIRC, and LIHC. (TIF 189 kb) Additional file 2: Figure S2. The impact of gene expression abundance on the measure of cooperation. The scatterplots containing the pair-wise correlation coefficient on the y-axis and the average expression of genes (log_2_ scale) on the x-axis. (TIF 316 kb) Additional file 3: Figure S3. Transcriptional abundances of TFs and non-TFs across cancers. TFs show comparable expression abundance with non-TFs in both normal and tumor. TFs are colored in red and non-TFs are colored in blue respectively. The expression value is log_2_ transformed. (TIF 193 kb) Additional file 4: Figure S4. Cooperation of TFs, non-TFs, TF-targets and metabolic genes in normal samples. TFs, non-TFs and TF targets showed similar cooperativity level in all types of matched normal samples. (TIF 181 kb) Additional file 5: Figure S5. Comparison of cooperation alterations between highly coordinated TFs in normal and those in tumor. (TIF 153 kb) Additional file 6: Figure S6. The correlation measures of recurrent TF pairs in normal and tumor. Normal samples are colored in dark blue while the tumor ones are colored in dark red. r_N_ and r_T_ denote the Spearman correlation coefficients in normal and tumor respectively. (TIF 1020 kb) Additional file 7: Table S1. Identified highly dyscooperated TFs of each cancer type. (XLSX 15 kb) Additional file 8: Figure S7. Comparison of expression alterations of IRF’s targets and other genes involved in RS. Each of the six plots illustrates the cumulative distribution function (cdf) of expression change in tumor relative to normal for IRF’s targets versus other genes involved in the GO term of response to stress (RS). The x-axis is the absolute value of log_2_ transformation of fold change. (TIF 16347 kb) Additional file 9: Figure S8. The source of TFs data sets. (TIF 112 kb) Additional file 10: Figure S9. Cut-offs for identifying highly dyscooperated TFs. (A) The distribution of the number the Z-transformed cooperativity alteration $\left({Z}_{C_{ij}^{T-N}}\right)$ greater than 3. (B) The distribution of the number the Z-transformed cooperativity alteration $\left({Z}_{C_{ij}^{T-N}}\right)$ less than -3. The cut-off for identifying highly disrupted TFs is shown by the vertical line for each cancer type. (TIF 23144 kb) Additional file 11: Table S2. Encode DNase-seq data used to filter motif hits. (XLSX 10 kb) Additional file 12: Table S3. List of IRF8’s targets and those involved in stress response in NSCLC. (XLSX 32 kb)
