# Supplementary figures and images for: Disrupted cooperation between transcription factors across diverse cancer types
Source: BMC Genomics. 2016 Aug 5;17:560. doi: 10.1186/s12864-016-2842-8 (PMC4975902; doi:10.1186/s12864-016-2842-8)

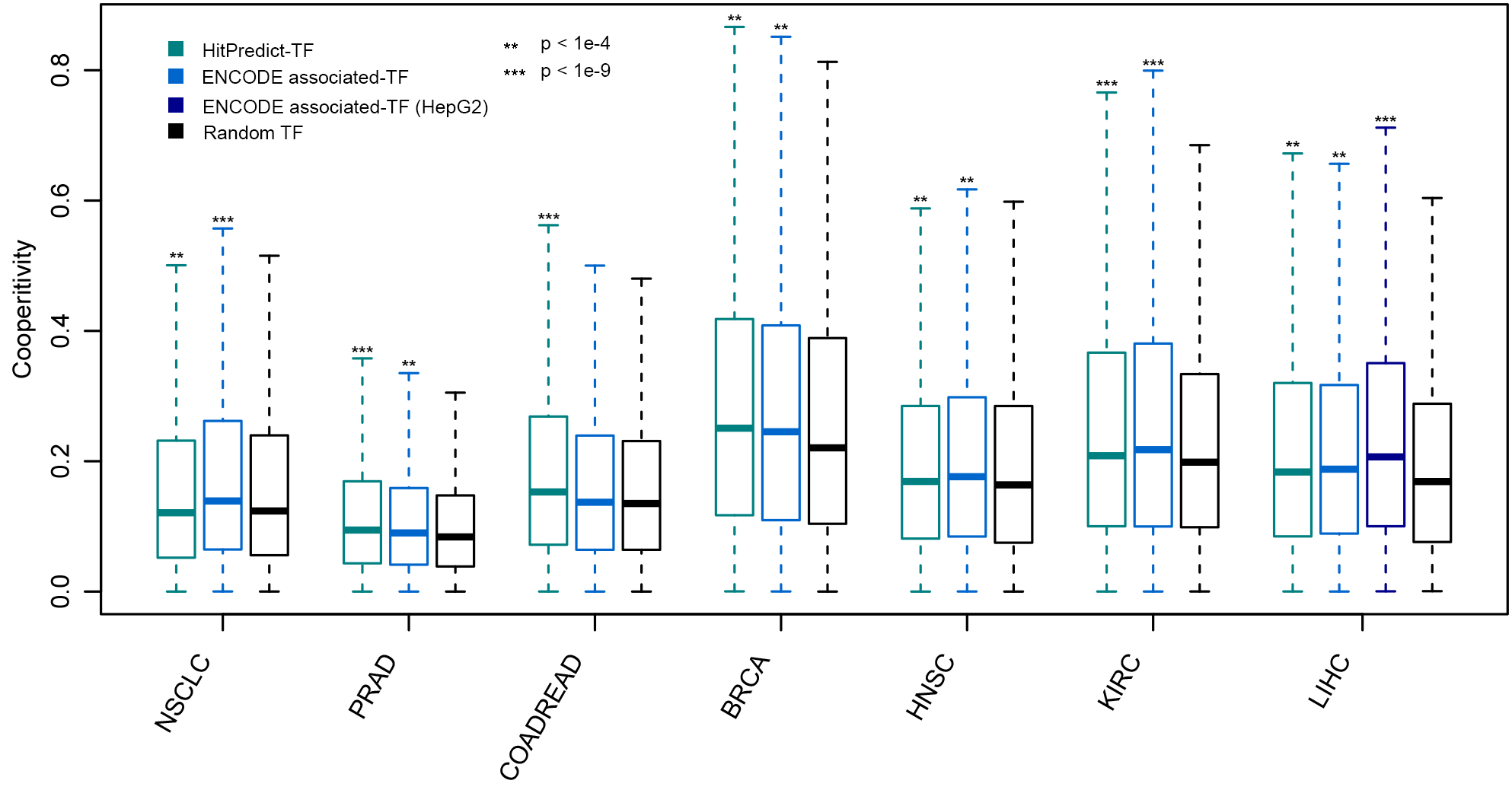

Supplement: Additional file 1: Figure S1. — The correlation between TF pairs from different datasets. The box plots illustrate the correlation (absolute value of Spearman correlation coefficient) between interacted TFs from HitPredict (HitPredict-TF), associated TFs and HepG2-specific associated TFs from ENCODE (ENCODE associated-TF), and the correlation of random TF pairs (random TF) in NSCLC, PRAD, COADREAD, BRCA, HNSC, KIRC, and LIHC. (TIF 189 kb) [file 12864_2016_2842_MOESM1_ESM.tif]

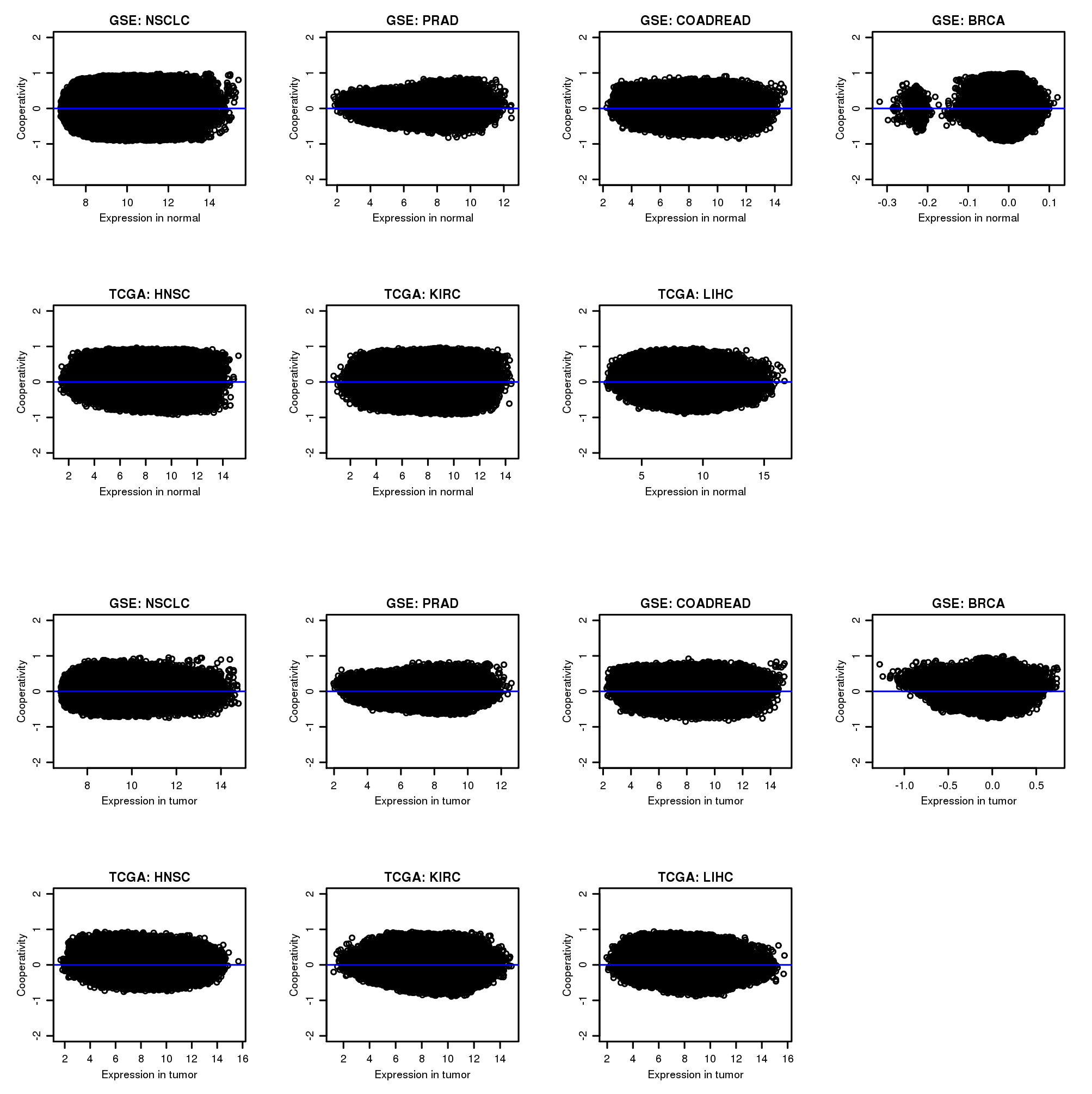

Supplement: Additional file 2: Figure S2. — The impact of gene expression abundance on the measure of cooperation. The scatterplots containing the pair-wise correlation coefficient on the y-axis and the average expression of genes (log2 scale) on the x-axis. (TIF 316 kb) [file 12864_2016_2842_MOESM2_ESM.tif]

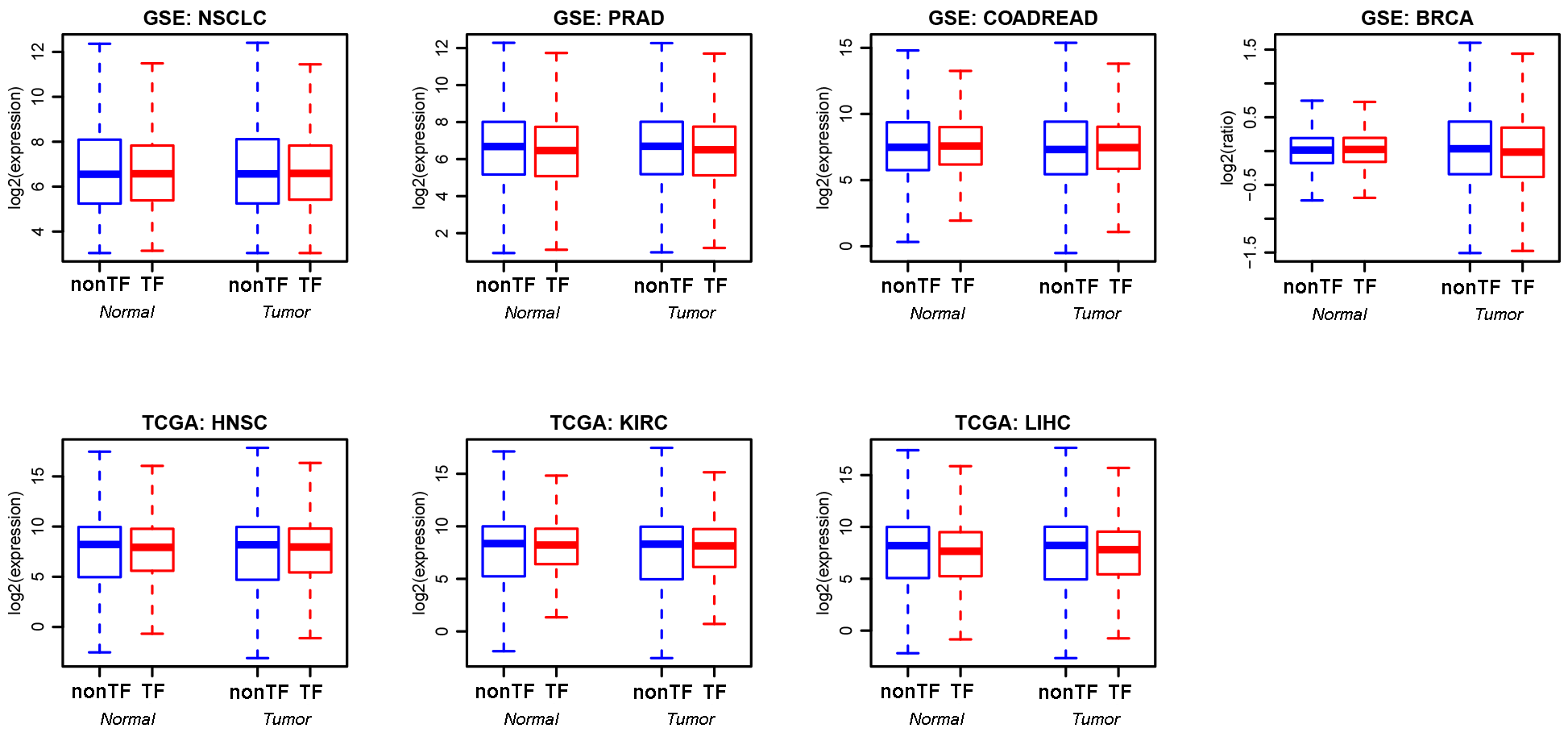

Supplement: Additional file 3: Figure S3. — Transcriptional abundances of TFs and non-TFs across cancers. TFs show comparable expression abundance with non-TFs in both normal and tumor. TFs are colored in red and non-TFs are colored in blue respectively. The expression value is log2 transformed. (TIF 193 kb) [file 12864_2016_2842_MOESM3_ESM.tif]

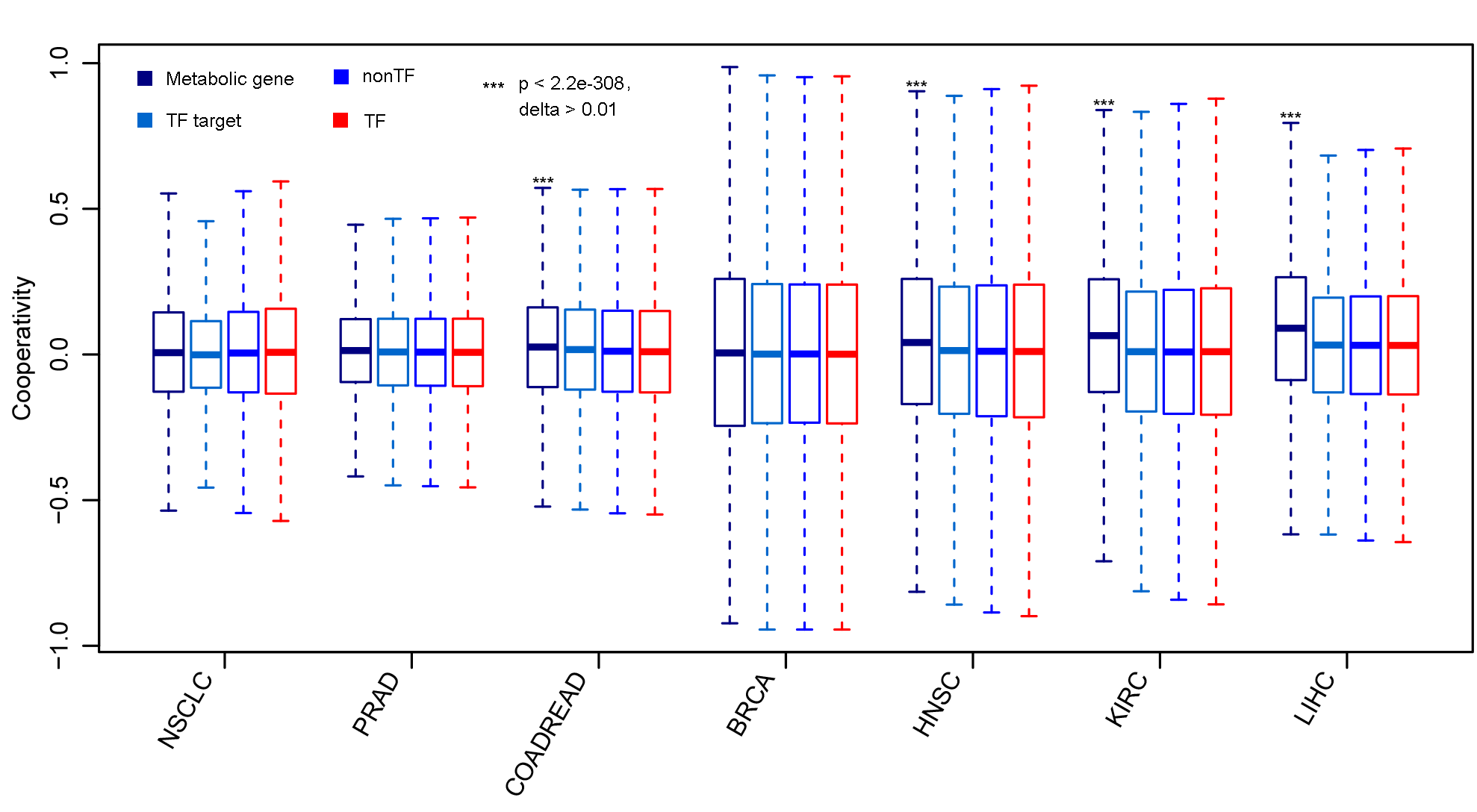

Supplement: Additional file 4: Figure S4. — Cooperation of TFs, non-TFs, TF-targets and metabolic genes in normal samples. TFs, non-TFs and TF targets showed similar cooperativity level in all types of matched normal samples. (TIF 181 kb) [file 12864_2016_2842_MOESM4_ESM.tif]

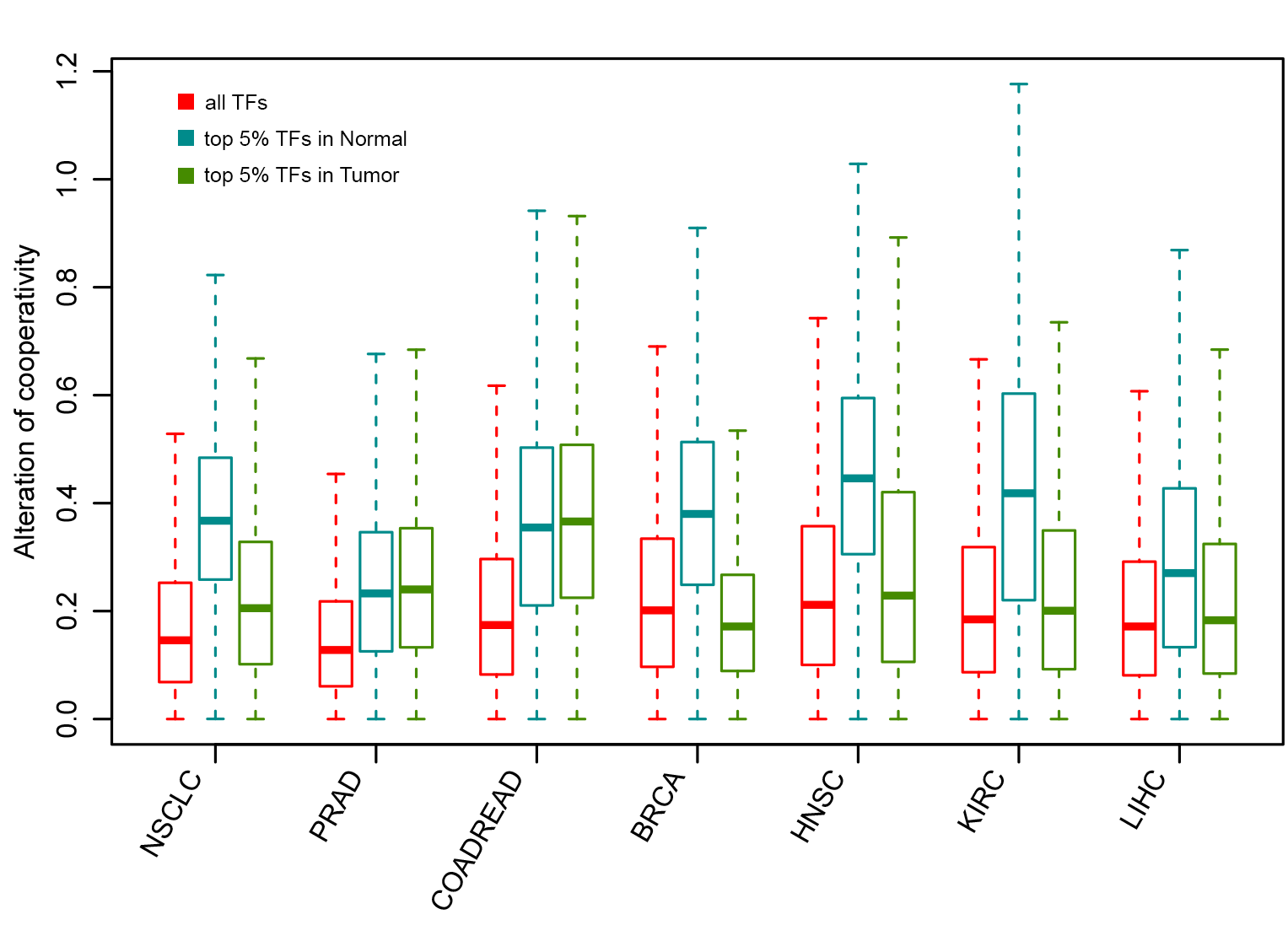

Supplement: Additional file 5: Figure S5. — Comparison of cooperation alterations between highly coordinated TFs in normal and those in tumor. (TIF 153 kb) [file 12864_2016_2842_MOESM5_ESM.tif]

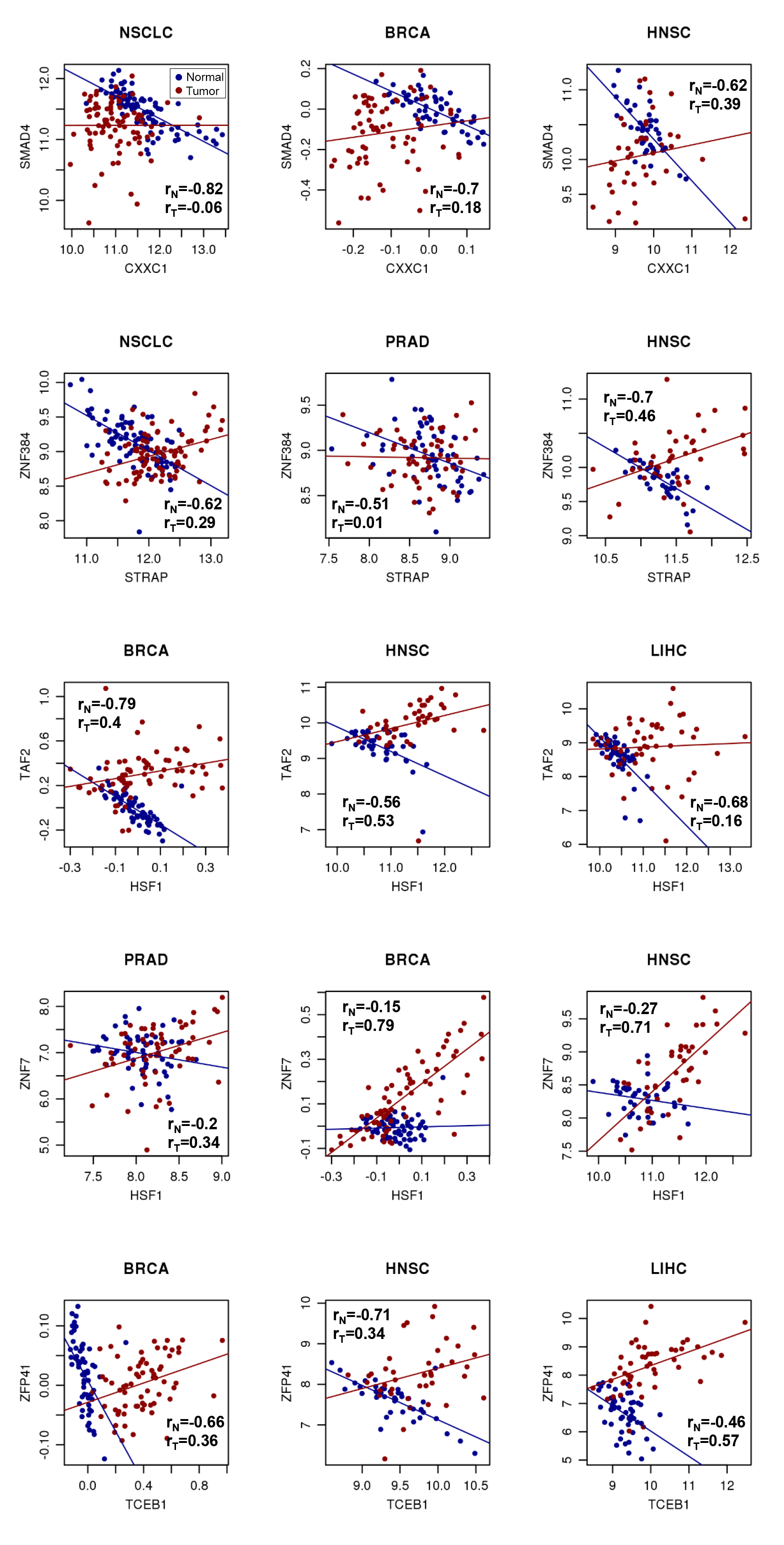

Supplement: Additional file 6: Figure S6. — The correlation measures of recurrent TF pairs in normal and tumor. Normal samples are colored in dark blue while the tumor ones are colored in dark red. rN and rT denote the Spearman correlation coefficients in normal and tumor respectively. (TIF 1020 kb) [file 12864_2016_2842_MOESM6_ESM.tif]

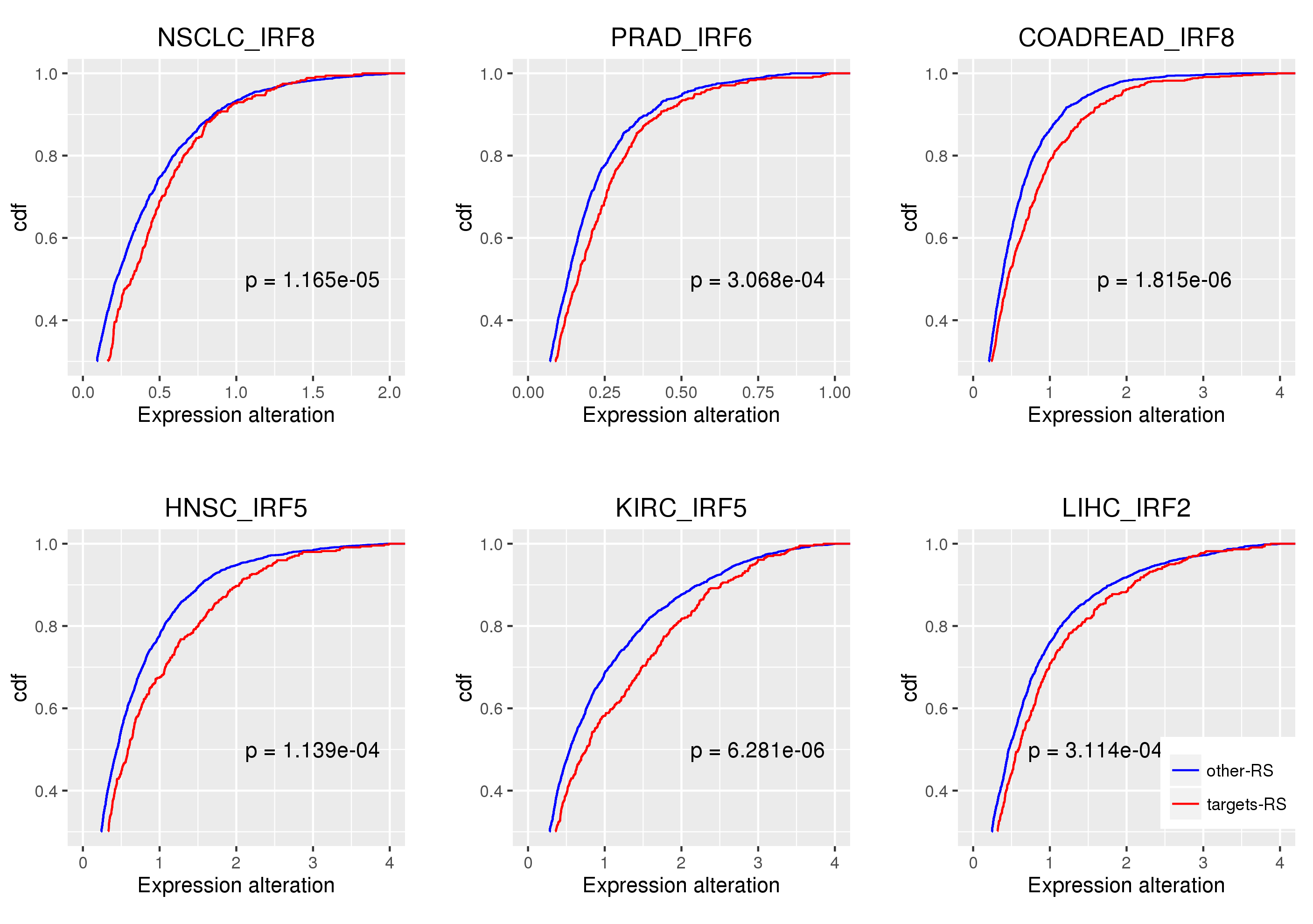

Supplement: Additional file 8: Figure S7. — Comparison of expression alterations of IRF’s targets and other genes involved in RS. Each of the six plots illustrates the cumulative distribution function (cdf) of expression change in tumor relative to normal for IRF’s targets versus other genes involved in the GO term of response to stress (RS). The x-axis is the absolute value of log2 transformation of fold change. (TIF 16347 kb) [file 12864_2016_2842_MOESM8_ESM.tif]

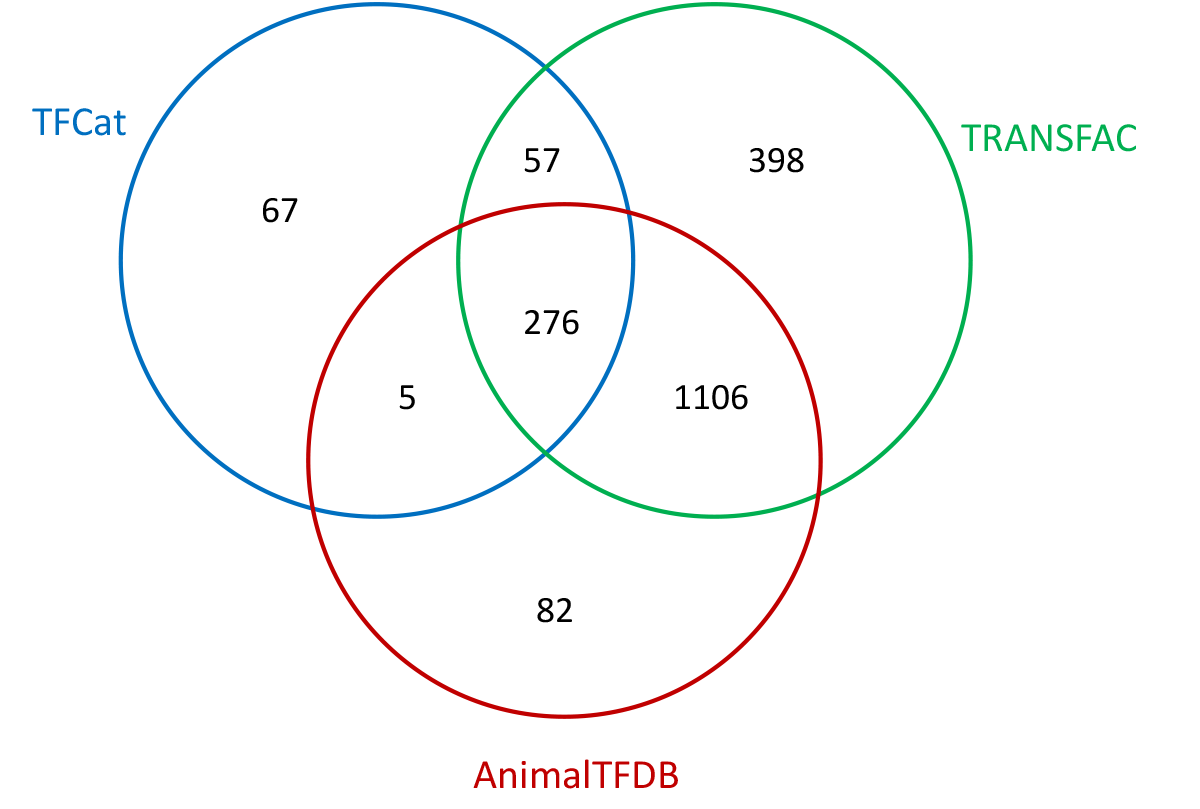

Supplement: Additional file 9: Figure S8. — The source of TFs data sets. (TIF 112 kb) [file 12864_2016_2842_MOESM9_ESM.tif]

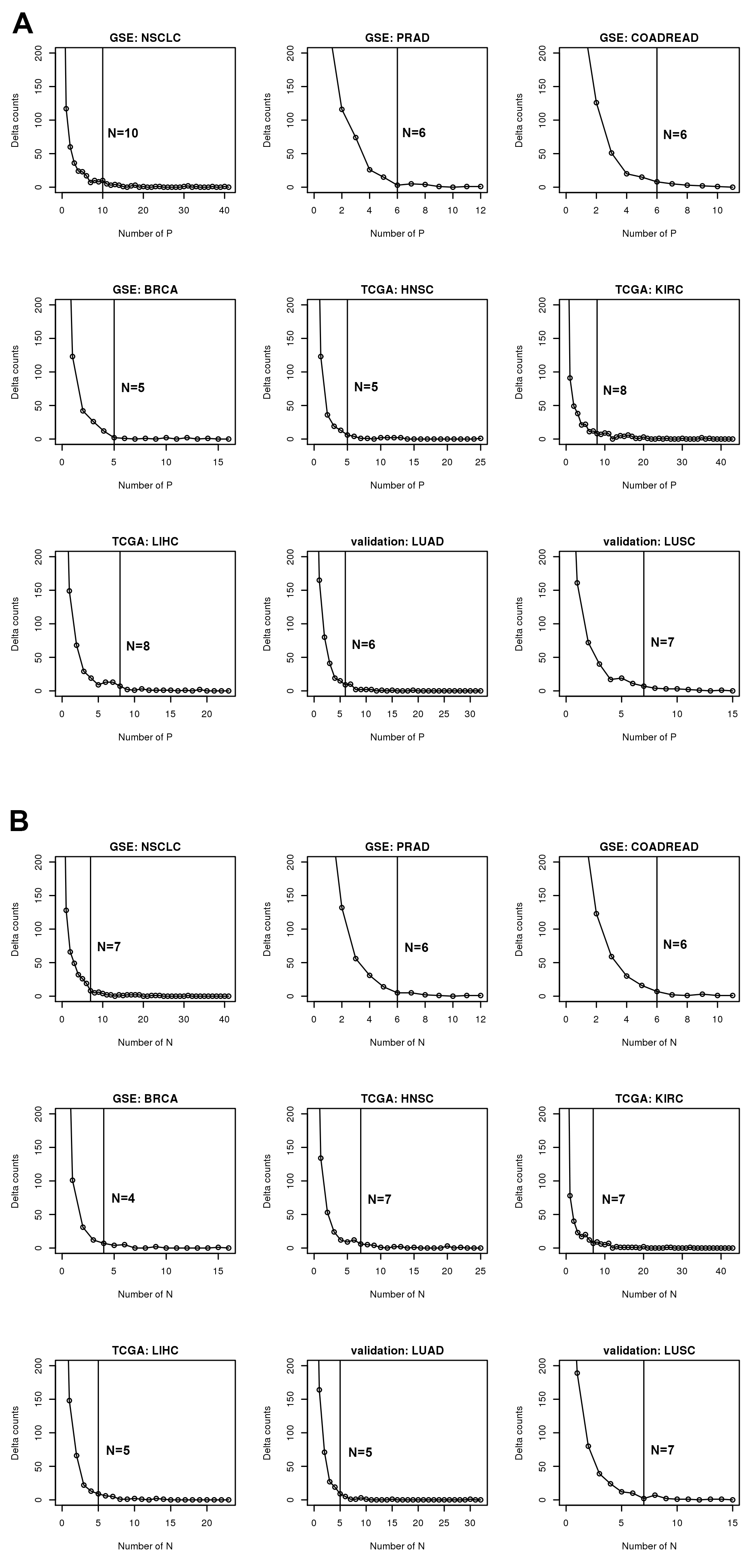

Supplement: Additional file 10: Figure S9. — Cut-offs for identifying highly dyscooperated TFs. (A) The distribution of the number the Z-transformed cooperativity alteration $\left({Z}_{C_{ij}^{T-N}}\right)$ZCijT−N greater than 3. (B) The distribution of the number the Z-transformed cooperativity alteration $\left({Z}_{C_{ij}^{T-N}}\right)$ZCijT−N less than -3. The cut-off for identifying highly disrupted TFs is shown by the vertical line for each cancer type. (TIF 23144 kb) [file 12864_2016_2842_MOESM10_ESM.tif]
